# First Identification of 12β-Deoxygonyautoxin 5 (12α-Gonyautoxinol 5) in the Cyanobacterium *Dolichospermum circinale* (TA04) and 12β-Deoxysaxitoxin (12α-Saxitoxinol) in *D. circinale* (TA04) and the Dinoflagellate *Alexandrium pacificum* (Group IV) (120518KureAC)

**DOI:** 10.3390/md20030166

**Published:** 2022-02-25

**Authors:** Michiru Akamatsu, Ryosuke Hirozumi, Yuko Cho, Yuta Kudo, Keiichi Konoki, Yasukatsu Oshima, Mari Yotsu-Yamashita

**Affiliations:** 1 Graduate School of Agricultural Science, Tohoku University, Sendai 980-8572, Japan; mg_rbbs9_0130@icloud.com (M.A.); ryousuke.hirozumi.t2@dc.tohoku.ac.jp (R.H.); yuko.cho.a4@tohoku.ac.jp (Y.C.); yuta.kudo.d5@tohoku.ac.jp (Y.K.); keiichi.konoki.b2@tohoku.ac.jp (K.K.); 2 Frontier Research Institute for Interdisciplinary Sciences, Tohoku University, Sendai 980-8578, Japan; 3 Graduate School of Life Sciences, Tohoku University, Sendai 980-8577, Japan; oshima.y3@gmail.com

**Keywords:** saxitoxin, *Dolichospermum circinale*, *Alexandrium pacificum*, paralytic shellfish toxins, 12β-deoxygonyautoxin 5 (12α-gonyautoxinol 5), 12β-deoxysaxitoxin (12α-saxitoxinol)

## Abstract

Saxitoxin and its analogues, paralytic shellfish toxins (PSTs), are potent and specific voltage-gated sodium channel blockers. These toxins are produced by some species of freshwater cyanobacteria and marine dinoflagellates. We previously identified several biosynthetic intermediates of PSTs, as well as new analogues, from such organisms and proposed the biosynthetic and metabolic pathways of PSTs. In this study, 12β-deoxygonyautoxin 5 (12α-gonyautoxinol 5 = gonyautoxin 5-12(*R*)-ol) was identified in the freshwater cyanobacterium, *Dolichospermum circinale* (TA04), and 12β-deoxysaxitoxin (12α-saxitoxinol = saxitoxin-12(*R*)-ol) was identified in the same cyanobacterium and in the marine dinoflagellate *Alexandrium pacificum* (Group IV) (120518KureAC) for the first time from natural sources. The authentic standards of these compounds and 12α-deoxygonyautoxin 5 (12β-gonyautoxinol 5 = gonyautoxin 5-12(*S*)-ol) were prepared by chemical derivatization from the major PSTs, C1/C2, produced in *D. circinale* (TA04). These standards were used to identify the deoxy analogues by comparing the retention times and MS/MS spectra using high-resolution LC-MS/MS. Biosynthetic or metabolic pathways for these analogues have also been proposed based on their structures. The identification of these compounds supports the α-oriented stereoselective oxidation at C12 in the biosynthetic pathway towards PSTs.

## 1. Introduction

**Figure 1:**
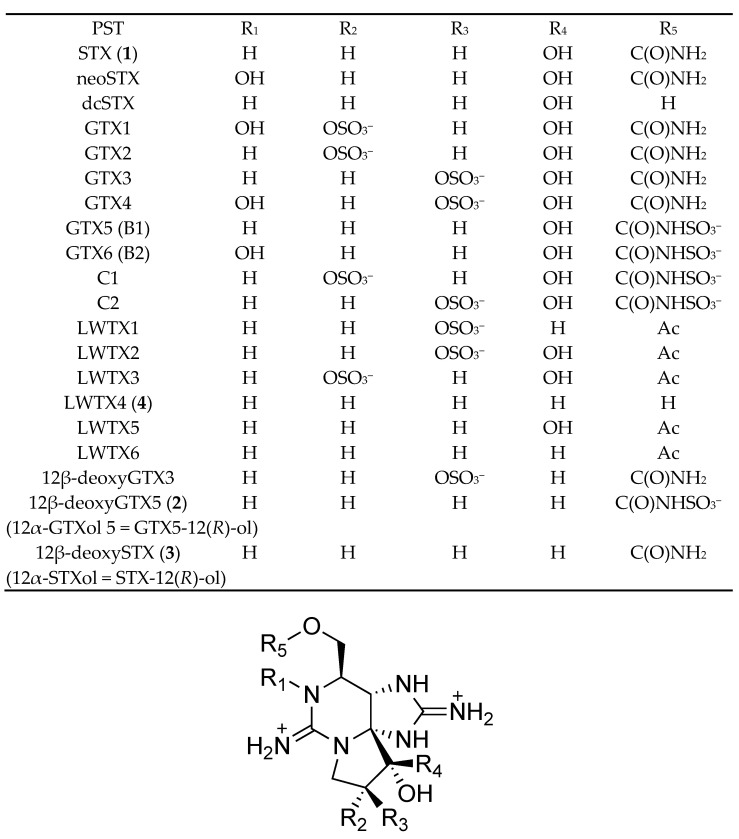
The structures of natural analogues of saxitoxin (STX, **1**). 12β-deoxyGTX5 (12α-GTXol 5) (**2**) and 12β-deoxySTX (12α-STXol) (**3**) are the analogues identified in natural sources for the first time in this study.

Saxitoxin (STX, **1**, Figure 1) is one of the most potent voltage-gated sodium channel (Na_v_) blockers [1]. More than 50 natural STX analogues, which are known as paralytic shellfish toxins (PST) [2,3], have been reported [4]. These toxins are produced by some species of freshwater cyanobacteria and marine dinoflagellates [5,6]. Due to their potent physiological effects and unique structures, chemical and biological aspects of STX and its analogues have been studied intensively by chemists and pharmacologists [7,8]. The first biosynthetic study of STX was a feeding experiment conducted by Shimizu et al. [9], in which stable isotope-labeled acetic acid and amino acids were used as essential substrates for PST-producing cyanobacteria and dinoflagellates. Next, Neilan’s group [10] discovered putative STX biosynthetic gene clusters (*sxt*) in the cyanobacterium, *Raphidopsis raciborskii* (formerly described as *Cylindrospermopsis raciborskii*) T3, and proposed a biosynthetic pathway different from that reported by Shimizu et al. [9]. The majority of the core set of 14 genes (*sxtA-sxtI*, *sxtP-sxtR*, *sxtS*, and *sxtU*) have commonly been identified in PST-producing cyanobacteria. Similarly, nuclear-encoded genes for STX in dinoflagellates were also discovered [11]. Subsequently, our group identified biosynthetic intermediates of STX, such as Int-A′, Int-C′2, 11-hydroxy Int-C′2, Int-E′, and a shunt compound, Cyclic-C′, in the PST-producing cyanobacterium *Dolichospermum circinale* (formerly described as *Anabaena circinalis*) (TA04), and the PST-producing dinoflagellate *Alexandrium catenella* (Group I) (formerly described as *Alexandrium tamarense*) (Axat-2), using LCMS with synthetic standards and by feeding experiments [12,13,14,15]. These findings allowed us to support the pathway proposed by Neilan’s group and propose a slightly revised biosynthetic pathway responsible for STX production based on the structures of these intermediates. We also reported that SxtA localizes to chloroplasts of toxic *A. catenella* (Group I)(Axat-2) [16]. Recently, Narayan’s group demonstrated the functions of several enzymes encoded in cyanobacterial PSTs’ biosynthetic gene clusters by heterologous expression [17,18,19,20,21]. They successfully expressed a polyketide-like synthase, SxtA, and enzymes that catalyze C-H hydroxylation, namely SxtT, SxtH, and GxtA. In addition, SxtSUL and SxtN from *R. raciborskii* T3 were indicated to act as *O*-sulfotransferase and *N*-sulfotransferase, respectively. SxtN was suggested to be implicated in the conversion from STX to gonyautoxin 5 (GTX5) (Figure 1). Neilan’s group also succeeded heterologous expression of SxtA [22].

Freshwater cyanobacteria and marine dinoflagellates are sources of various PST analogues [23] as are shellfish [24,25]. Six STX analogues, namely LWTX1-6, were isolated from the freshwater cyanobacterium *Microseira wollei* (formerly described as *Lyngbya wollei*), and their corresponding chemical structures were determined by NMR spectroscopic analysis by Onodera et al. (Figure 1) [26]. Three of these analogues have an α-hydroxyl group instead of a hydrated ketone at C12 in STX, and five of them contain an *O*-acetyl moiety at C13 instead of the *O*-carbamoyl moiety in STX. Among them, LWTX-4 (12β-deoxy-dcSTX = 12α-dcSTXol) (**4**) was also identified in the marine dinoflagellate *Alexandrium pacificum* (Group IV) (formerly described as *Alexandrium catenella*) (120518KureAC) by our group [27]. Lim et al. [28] reported 12-deoxyGTX4 in *A. minutum* without the identification of C12 stereochemistry. In our previous study, we screened for novel PST-related compounds using LCMS and identified 12β-deoxygonyautoxin 3 (12β-deoxyGTX3) (Figure 1) in *D. circinale* (TA04) extract [29].

In this study, further screening for new STX analogues was performed to collect more information on PST biosynthetic and metabolic pathways. As a result, two new STX analogues, 12β-deoxygonyautoxin 5 (12β-deoxyGTX5) (**2**) (12α-gonyautoxinol 5 = GTX5-12(*R*)-ol) and 12β-deoxySTX (**3**) (12α-saxitoxinol = STX-12(*R*)-ol), were identified in the cyanobacterium *D. circinale* (TA04), and **3** was also identified in the marine dinoflagellate *A. pacificum* (Group IV) (120518KureAC). The hypothetical biosynthetic or metabolic pathways of these compounds are also discussed.

## 2. Results

### 2.1. Screening for New PSTs in the PST-Producing Cyanobacterium D. circinale (TA04) and the Dinoflagellate A. pacificum (Group IV) (120518KureAC)

Screening for new STX analogues was performed on the semi-purified extracts from the cyanobacterium *D. circinale* (TA04) and the dinoflagellate *A. pacificum* (Group IV) (120518KureAC) (see Section 4.5 and Section 4.6 for a description of the preparation of the semi-purified cell extracts) using high resolution (HR)-LCMS, with both hydrophilic interaction liquid chromatography (HILIC) [27] and reversed phase (RP) separations in the positive mode. The screening using RP separation resulted in the identification of the unknown peaks in each organism. In *D. circinale* (TA04), an unknown peak detected at 13.2 min on the extracted ion chromatogram (EIC) of RP-LCMS detected at *m/z* 364.1034 ± 0.02 ([M + H]^+^; C_10_H_18_N_7_O_6_S) showed HRMS ([M + H]^+^ *m/z* 364.0985) under RP conditions, which was supposed to be the deoxy analogue of GTX5 ([M + H]^+^; C_10_H_18_N_7_O_7_S). Since some C12-hydrated ketone-reduced PST analogues have been previously identified in cyanobacteria as described above (LWTX1, LWTX4 (**4**), LWTX6, and 12β-deoxyGTX3 in Figure 1), this compound was supposed to be 12-deoxyGTX5.

Another unknown peak was detected in *D. circinale* (TA04) at 12.9 min and also in *A. pacificum* (Group IV) (120518KureAC) at 12.8 min on the EIC of RP-LCMS detected at *m/z* 284.1466 ± 0.02 ([M + H]^+^; C_10_H_18_N_7_O_3_); HRMS ([M + H]^+^ *m/z* 284.1443 for TA04, and *m/z* 284.1463 for 120518KureAC), which was supposed to be the deoxy analogue of STX ([M + H]^+^ C_10_H_18_N_7_O_4_).

### 2.2. Chemical Preparation of Authentic 12β-DeoxyGTX5 *(**2**)*, 12β-DeoxySTX *(**3**)*, and 12α-DeoxyGTX5 *(**5**)*

**Figure 2:**
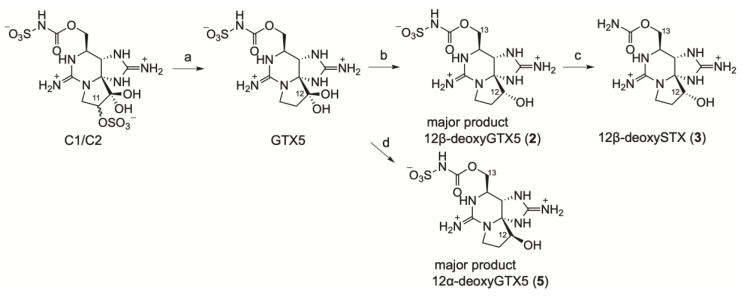
Preparation of compounds **2**, **3**, and **5** from C1/C2: (**a**) 2-mercaptoethanol/100 mM sodium acetate buffer (pH 5.2) (1:1, *v*/*v*), 50 °C, 2 h. [30]; (**b**) NaBH_4_/water, 0 °C, 30 min. [31]; (**c**) 0.13 M HCl, 100 °C, 15 min; and (**d**) NaBH_4_ (quick addition)/MeOH, 55 °C, 60 min [31].

For further identification, authentic 12β-deoxyGTX5 (**2**), 12β-deoxySTX (**3**), and 12α-deoxyGTX5 (**5**) were synthesized from a mixture of C1 and C2 (C1/C2), which was obtained from *D. circinale* (TA04) as the main PST, as shown in Figure 2. The sulfate group at C11 in C1/C2 (43 µg, 92 nmol) was desulfated with 2-mercaptoethanol to produce GTX5 according to the method reported by Watanabe et al. [30], and the hydrated ketone at C12 in GTX5 was reduced with NaBH_4_ in water at 0 °C using the method reported by Koehn et al. [31] to the diastereomer mixture of 12β-deoxyGTX5 (**2**) and 12α-deoxyGTX5 (**5**) (approximate ratio 10:1, mol/mol based on LCMS area, approximate yield of **2** for two steps after purification: 15 µg, 40 nmol, 43%). The mixture of **2** and **5** (approximate ratio 4:1, mol/mol, based on LCMS area) was also prepared from GTX5 in MeOH by adding NaBH_4_ relatively slowly at 55 °C [31] for the standard of LCMS analysis. In addition, 12α-deoxyGTX5 (**5**) was prepared from GTX5 by adding NaBH_4_ quickly in MeOH solution of GTX5 at 55 °C; this yielded the mixture of **2** and **5** (approximate ratio 1:2, mol/mol, based on LCMS area). After purification, the approximate yield of **5** from 55 µg of C1/C2 was 10 µg, 27 nmol, 23% (mol/mol). For the same purpose, the mixture of 12β-deoxySTX (**3**) and 12α-deoxySTX (approximate ratio 10:1, based on LCMS area) was also prepared from the mixture of **2** and **5** (approximate ratio 10:1, based on LCMS area). Continuously, 12β-deoxySTX (**3**) was obtained from purified **2** by acid hydrolysis of *N*-sulfate [30] (quant.). The amount of 12α-deoxySTX was too small to be identified by ^1^H NMR spectroscopy, but production of this epimer as the minor component was supposed according to the previous report [31].

**Figure 3:**
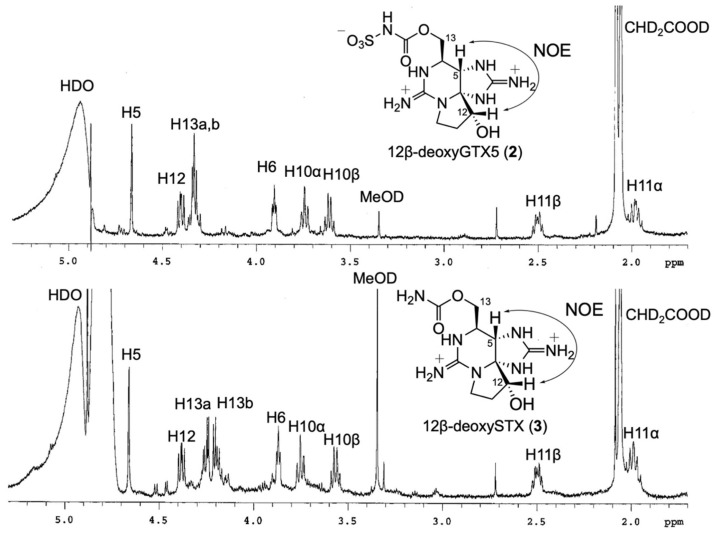
^1^H NMR spectra and observed key NOEs of synthetic 12β-deoxyGTX5 (**2**) and 12β-deoxySTX (**3**). A 600 MHz, CD_3_COOD-D_2_O (4:96, *v*/*v*), HDO signal was suppressed. The signal of CHD_2_COOD (2.06 ppm) was used as internal reference.

**Figure 4:**
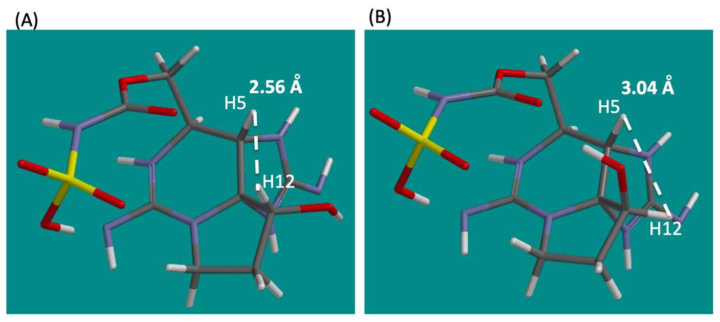
Stable conformers of (**A**) 12β-deoxyGTX5 (**2**) and (**B**) 12α-deoxyGTX5 (**5**) shown with the distances between H5 and H12. The models were calculated using Spartan‘18 with molecular mechanics, Merck Molecular Forcefield (MMFF).

After purification with activated charcoal, 12β-deoxyGTX5 (**2**) (HRMS ([M + H]^+^ *m/z* 364.1028 C_10_H_18_N_7_O_6_S, Δ 1.6 ppm, Figure S1), 12β-deoxySTX (**3**) (HRMS ([M + H]^+^ *m/z* 284.1464 C_10_H_18_N_7_O_3_, Δ 0.4 ppm, Figure S2) and 12α-deoxyGTX5 (**5**) (HRMS ([M + H]^+^ *m/z* 364.1024 C_10_H_18_N_7_O_6_S, Δ 0.3 ppm, Figure S3) were analyzed using ^1^H NMR spectroscopy to confirm their structures, including the stereochemistry at C12 for **2** and **3** (Figure 3). The ^1^H NMR signals of **2**, **3**, and **5** (Table 1) were assigned based on COSY and/or TOCSY correlations (Figures S4–S8) and compared with ^1^H NMR data of LWTX-4 (12β-deoxy-dcSTX) (**4**) and other similar analogues reported by Onodera et al. [26]. The chemical shifts of the ^1^H NMR spectra of **2** and **3** are close to those of **4**. The chemical shifts of H6 and H13a, b of **2** and **3** were more than 0.2 ppm shifted from those of **4**, which could be explained by the structural difference of the 13-*O*-substituents. The β-orientation of H12 of **2** and **3** was confirmed by the observed positive NOEs between H5 and H12 by measuring the NOESY 1D spectra (Figures S9 and S10), and the almost identical multiplicity of H12 in **2**, **3** (dd 7.6, 11.8 Hz), and **4** (dd 7.6, 11.6 Hz) [26]. In addition, ^1^H NMR signals of 12α-deoxyGTX5 (**5**) were compared with those of β-saxitoxinol (12α-deoxySTX) reported by Koehn et al. [31] The similar multiplicity and *J* value of H12 in 12α-deoxyGTX5 (**5**) (d 4.7 Hz) to those of H12 in β-saxitoxinol (d 4.6 Hz) supported the stereochemistry of C12 in **5**. NOESY1D experiments for 12α-deoxyGTX5 (**5**) were also conducted as described above, and no significant NOE was detected between H5 and H12. Energy-minimized molecular models of **2** and **5,** which were calculated using Spartan‘18 (Wavefunction, Irvine, USA), with molecular mechanics, Merck Molecular Forcefield (MMFF), estimated that the distances between H5 and H12 in **2** and **5** were 2.56 and 3.04 Å, respectively (Figure 4). These data all supported stereochemical assignment at C12 in synthetic **2**, **3**, and **5**.

### 2.3. Identification of 12β-DeoxyGTX5 *(**2**)* in D. circinale (TA04)

**Figure 5:**
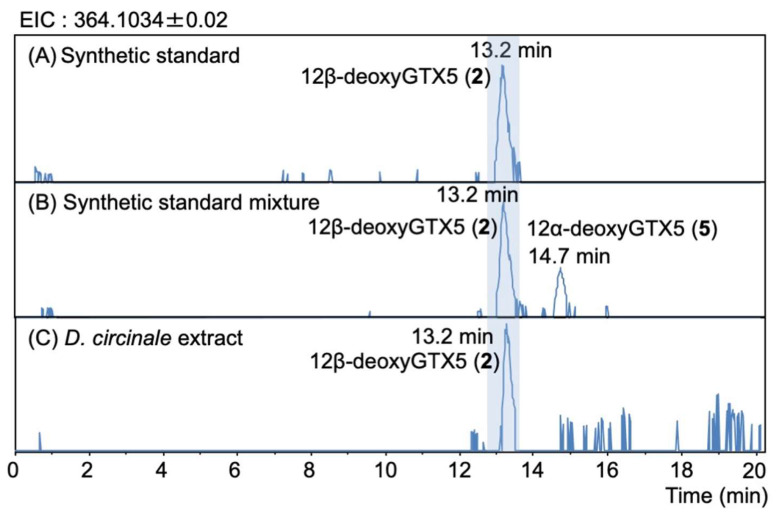
HR-LCMS chromatograms (EIC *m/z* 364.1034 ± 0.02) of (**A**) synthetic 12β-deoxyGTX5 (**2**) standard, (**B**) synthetic standard mixture of 12β-deoxyGTX5 (**2**) and 12α-deoxyGTX5 (**5**), and (**C**) semi-purified *D. circinale* (TA04) extract. LC was performed under RP condition (see, Methods and Materials Section).

**Figure 6:**
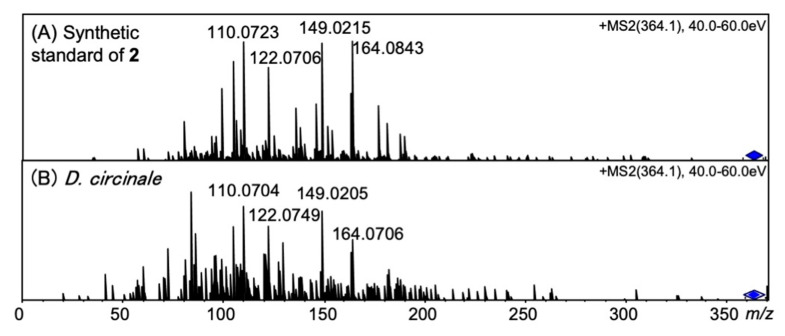
MS/MS spectra of (**A**) synthetic 12β-deoxyGTX5 (**2**) standard and (**B**) **2** in semi-purified *D. circinale* (TA04) extract.

The peaks of synthetic 12β-deoxyGTX5 (**2**) (13.2 min) and 12α-deoxyGTX5 (**5**) (14.7 min) were separated under RP-LCMS conditions (Figure 5A,B). The unknown peak detected in *D. circinale* (TA04) on the EIC at *m/z* 364.1034 ± 0.02 at 13.2 min was identified as 12β-deoxyGTX5 (**2**) by comparing its LCMS (Figure 5C) and the HR-MS detected at *m/z* 364.0985 (calcd. *m/z* 364.1034) for this peak (Figure S11). LC-MS/MS spectra (Figure 6A,B) with that of authentic **2** also supported the identification. The HR-MS of the major fragment ions detected at *m/z* 149.0205, 122.0749, and 110.0704 for 12β-deoxyGTX5 (**2**) in *D. circinale* (Figure 6B) were almost identical with those detected for synthetic 12β-deoxyGTX5 (**2**) (Figure 6A) (the differences were within 0.0043 Da), supporting the identification of **2** in *D. circinale*.

### 2.4. Identification of 12β-DeoxySTX *(**3**)* (12α-STXol) in A. pacificum (Group IV) (120518KureAC) and D. circinale (TA04)

**Figure 7:**
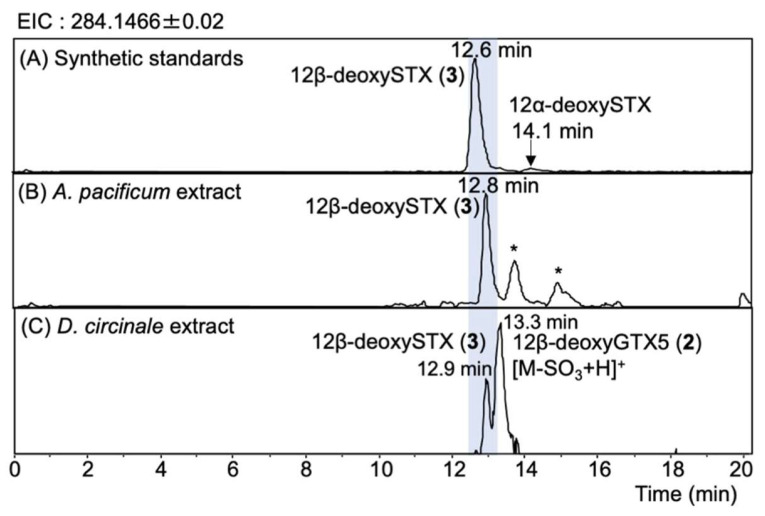
HR-LCMS chromatograms (extracted ion chromatogram (EIC) *m/z* 284.1466 ± 0.02) of (**A**) the mixture of synthetic 12β-deoxySTX (**3**) and 12α-deoxySTX standard, (**B**) semi-purified *A. pacificum* (Group IV) (120518KureAC) extract, and (**C**) semi-purified *D. circinale* (TA04) extract. LC was performed under RP conditions (see, Methods and Materials Section). * HR-MS for these peaks were more than 0.01 Da different from 284.1466.

**Figure 8:**
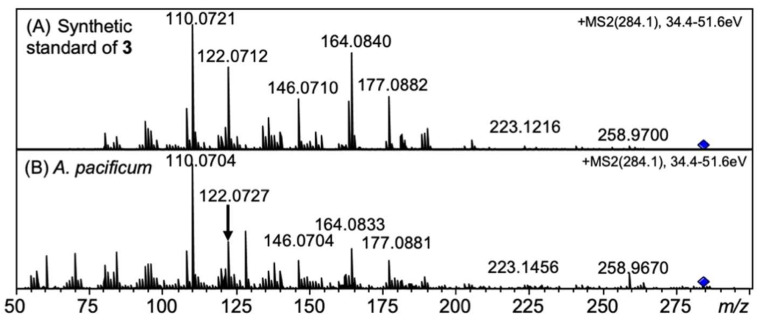
MS/MS spectra of (**A**) synthetic 12β-deoxySTX (**3**) standard and (**B**) 12β-deoxySTX (**3**) in semi-purified *A. pacificum* (Group IV) (120518KureAC) extract.

The peaks of synthetic 12β-deoxySTX (**3**) and 12α-deoxySTX standards were observed at 12.6 min and 14.1 min, respectively, on the EIC at *m/z* 284.1466 ± 0.02 of RP-LCMS (Figure 7A). The retention times of the unknown peak detected at 12.8 min in *A. pacificum* (Group IV) (120518KureAC) (Figure 7B) and at 12.9 min in *D. circinale* (TA04) (Figure 7C) were close to that of synthetic **3** (12.6 min), suggesting that these peaks correspond to 12β-deoxySTX (**3**). The HR-MS detected at *m/z* 284.1443 in *D. circinale* (TA04) and at *m/z* 284.1463 in *A. pacificum* (Group IV) (120518KureAC) (calcd. *m/z* 284.1466) for these peaks (Figures S12 and S13) supported identification. The presence of 12β-deoxySTX (**3**) in *A. pacificum* was further confirmed by comparing the LC-MS/MS spectrum with that of authentic **3** (Figure 8). The HR-MS of the major fragment ions detected in the MS/MS spectrum of **3** in *A. pacificum* (Figure 8B) were almost identical with those detected for synthetic 3 (Figure 8A) (the differences were within 0.002 Da), supporting identification of **3** in this species. The potential 12β-deoxySTX (**3**) peak detected in semi-purified *D. circinale* (TA04) extract has not been confirmed using the MS/MS spectrum because of the low intensity of this peak. The peak detected at 13.3 min in *D. circinale* (TA04) extract (Figure 7C) was interpreted as the desulfated 12β-deoxyGTX5 (**2**) ion ([M-SO_3_ + H]^+^) because of the close retention time to that of **2** (13.2 min, Figure 5C).

## 3. Discussion

In this study, 12β-deoxyGTX5 (**2**) was identified in the cyanobacterium *D. circinale* (TA04), and 12β-deoxySTX (**3**) was identified in the same cyanobacterium, as well as in the dinoflagellate *A. pacificum* (Group IV)(120518KureAC) by comparisons with the synthetically prepared **2**, **3**, and 12α-deoxyGTX5 (**5**) standards using LCMS and LC-MS/MS. On the other hand, 12α-deoxyGTX5 (**5**) and 12α-deoxySTX were not detected in these organisms. Koehn et al. [31] previously reported the chemical preparation of 12β-deoxySTX (**3**) (α-saxitoxinol) from STX, and Lukowski et al. [18] used synthetic **3** as the SxtN substrate to produce **2** (as detailed below). However, before the present study, **2** and **3** were not identified in natural sources, including PST-producing organisms. The other 12β-deoxy type PST analogue, 12β-deoxy-dcSTX (**4**) (LWTX-4) (Figure 1), was previously reported by us in *A. catenella* (Group I) (described as *A. tamarense* in the literature) (Axat-2) [27], and the cyanobacterium *Microseira wollei* [26]. LWTX-1 and LWTX-5, which are 13-*O*-acetate analogues, were also found in *M. wollei* [26], and we also previously reported finding 12β-deoxyGTX3 in *D. circinale* (TA04) [29]. Identifying these compounds supports the α-oriented stereoselective enzymatic oxidation at C12 in the biosynthetic pathway of PSTs.

The abundance ratios of 12β-deoxyGTX5 (**2**), 12β-deoxySTX (**3**), and 12β-deoxy-dcSTX (**4**) in *D. circinale* (TA04) cells were estimated to be 0.3%, 0.3%, and 1.1% (mol/mol) of C1/C2, respectively, based on the HR-LCMS Q1 scan peak area (Figure S14). In *A. pacificum* (120518KureAC), an abundance ratio of 12β-deoxySTX (**3**) was estimated to be 60% (mol/mol) of 12β-deoxy-dcSTX (**4**) and 1.2% (mol/mol) of C1/C2 in this study. We previously reported the intracellular PST content, including biosynthetic intermediates and a shunt product in this species [27]. In that study, the abundance ratio of **4** was 2.1% (mol/mol) of C2. C1/C2 are the major PST analogues in both organisms. The results suggest that both **2** and **3** are minor PST components in these species.

**Figure 9:**
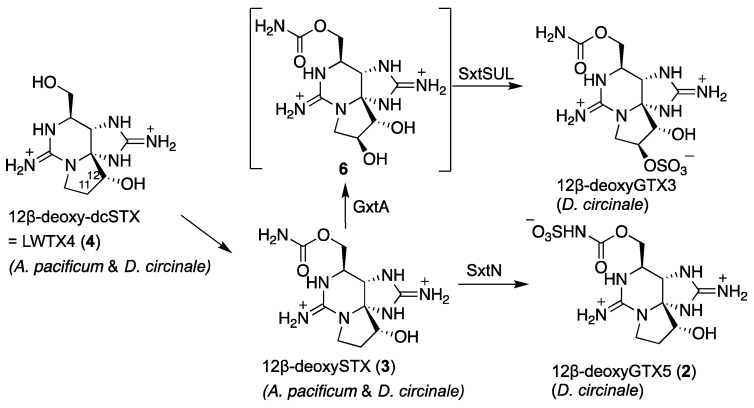
Possible biosynthetic routes from **4** to **2**, **3**, and 12β-deoxyGTX3 [29]. The predicted intermediate **6** has not been identified in a natural source, including PST-producing organisms.

Lukowski et al. [18] functionally expressed GxtA, Rieske oxygenase which is involved in β-hydroxylation at C11, and SxtSUL, *O*-sulfotransferase of 11β-hydroxyl STX, and SxtN, which is functionalized as *N*-sulfotransferase of the carbamoyl group in STX. SxtSUL was obtained from the cyanobacterium *Microseira wollei*, and SxtN came from *Aphanizomenon* sp. NH-5. Since we identified 12β-deoxyGTX5 (**2**) and 12β-deoxySTX (**3**) in *D. circinale* (TA04) in this study, a homologous enzyme with SxtN in *D. circinale* (TA04) should have a similar functionality to that of *M. wollei*. A possible biosynthetic route from **4** to **2**, **3**, and 12β-deoxyGTX3 which was previously found in *D. circinale* (TA04), is proposed in Figure 9 although the predicted compound **5** (11β-hydroxy-12β-deoxySTX) has not yet been identified. GxtA, SxtSUL, and SxtN were predicted to be involved in these reactions. The presence of **3** (in this study) and **4** in *Dolichospermum circinale* (TA04) [26] and *Alexandrium pacificum* (120518KureAC) [27] suggested the functional similarity of the enzyme that catalyzes *O*-carbamoylation of **4** in these organisms. Further biochemical and analytical studies are needed to compare similar biosynthetic reactions between cyanobacteria and dinoflagellates.

## 4. Methods and Materials

### 4.1. General Information

The reagents were purchased from Merck KGaA (Darmstadt, Germany), FUJIFILM Wako Pure Chemical Industries, Ltd. (Osaka, Japan), Tokyo Chemical Industry Co., Ltd. (Tokyo, Japan), and Nacalai Tesque, Inc. (Kyoto, Japan). LCMS-grade methanol (Kanto Chemical Co., Ltd., Tokyo, Japan) was used for HR-LCMS. Distilled and purified water (MilliQ) by Simplicity UV (Merck Millipore Corporation, Billerica, MA, USA) was used for all experiments. NMR spectra were recorded in micro-bottom tubes (Shigemi, Hachioji, Japan) at 20 °C with an Agilent 600 MHz NMR spectrometer (Agilent Technologies, Inc., Santa Clara, CA, USA) and a Bruker AVANCE III 600 spectrometer (Bruker, Billerica, MA, USA) with a 5 mm CryoProbe with CD_3_COOD-D_2_O (4:96, *v/v*). The spectra were referenced to CHD_2_COOD signals with resonances at δ_H_ = 2.06 ppm. HR-LCMS was performed using a micrOTOF-Q II (ESI, Q-TOF) (Bruker Daltonics Inc., Billerica, MA, USA).

### 4.2. Preparation of 12β-DeoxyGTX5 *(**2**)* and 12β-DeoxySTX *(**3**)* from C1/C2

The C1 and C2 mixture (C1/C2, 47 µg by LCMS) was purified from the *D. circinale* (TA04) cultures (175 mL) by chromatography on activated charcoal (FUJIFILM Wako Pure Chemical Industries) and Bio-gel P2 (BioRad, Hercules, CA, USA). C1/C2 was subsequently transformed into GTX5 by reacting it with 2-mercaptoethanol (0.1 mL) in 100 mM sodium acetate buffer (pH 5.2) (0.1 mL) at 50 °C for 2 h as reported by Watanabe et al. [30]. The resulting GTX5 (not quantified) was purified by chromatography on activated charcoal. Next, GTX5 was lyophilized and dissolved in water (0.2 mL). The resulting solution was transferred to another microtube containing 3.2 mg NaBH_4_ powder, which was placed on ice. The mixture was kept at 0 °C for 30 min [31]. After the reaction, the resultant solution was neutralized to pH 7–8 with 0.5 M AcOH, then applied to an activated charcoal column (0.8 mL). The mixture was eluted from the activated charcoal column with AcOH-EtOH-H_2_O 5:50:45 (*v/v/v*) (2.4 mL) after washing with water (0.8 mL). The production of 12β-deoxyGTX5 (**2**) with trace amount of 12α-deoxyGTX5 (**5**) (approximately 10:1, mol/mol) was confirmed by LCMS analysis. 12β-deoxyGTX5 (**2**) (approx. 20 µg, estimated by ^1^H NMR) was obtained after HPLC purification with an InertSustain AQ-C18 (4.6 i.d. × 250 mm, 5 μm, GL Sciences, Japan) column with HCOOH-H_2_O 0.1:100 (*v/v*) as the mobile phase. The flow rate was 0.2 mL/min. The injected volume for purification was 50 µL, and the temprature was 20 °C. The yield of pure **2** from C1/C2 was approximately 56% (mol/mol).

Another portion of purified 12β-deoxyGTX5 (**2**) (approx. 40 µg) was dissolved in 0.13 M HCl aq. (0.2 mL) and heated at 100 °C for 15 min under a N_2_ atmosphere. After purification with charcoal as described above, 12β-deoxySTX (**3**) (approx. 20 µg, estimated by ^1^H NMR) was obtained in an almost pure form.

### 4.3. Preparation of the Mixture of 12α/β-DeoxyGTX5 *(**2**, **5**)* from GTX5 for LCMS Analysis

GTX5 (1 µg) prepared similarly from C1/C2 as described above was dissolved in MeOH (0.2 mL) and reacted with NaBH_4_ (3.2 mg) at 55 °C for 60 min [31]. After the reaction, the resultant solution was neutralized to pH 7–8 with 0.5 M AcOH, then applied to an activated charcoal column (0.5 mL). 12α/β-deoxyGTX5 (ratio 4:1, mol/mol) was eluted, as described above, and used for LCMS analysis.

### 4.4. Preparation of 12α-DeoxyGTX5 *(**5**)* for NMR Analysis

12α-deoxyGTX5 (**5**) was prepared by adding NaBH_4_ quickly into the MeOH solution of GTX5 (prepared from 55 µg of C1/C2, as described above) at 55 °C. After keeping the reaction for 1 h, the ratio of **2** and **5** was estimated to be approximately 1:2, mol/mol, based on LCMS area. The resultant solution was treated with an activated charcoal column (0.8 mL) as described above, and then applied to HPLC purification with an InertSustain AQ-C18 (4.6 i.d. × 250 mm, 5 μm, GL Sciences, Japan) column and the mobile phase, HCOOH-H_2_O 0.1:100 (*v/v*). The flow rate was 0.2 mL/min. The injected volume for purification was 50 µL, and the temperature was 20 °C. 12α-deoxyGTX5 (**5**) (10 µg, 27 nmol) was obtained in almost pure form. The yield of **5** from C1/C2 (55 µg) was approximately 23% (mol/mol).

### 4.5. Harvest and Preparation of D. circinale (TA04) Cell Extract

The toxic strain of the freshwater cyanobacterium *D. circinale* used in this study is a nonaxenic strain TA04. The field sample of *D. circinale* was collected at the Tullaroop reservoir, Victoria, Australia, and the TA04 strain was one of the single-trichome isolates prepared by Negri et al. in 1993 [32]. *D. circinale* (TA04) was provided by Dr. Susan Blackburn, CSIRO, Australia, and cultured in CB’ medium (Bicine instead of Tris (hydroxymethyl) aminomethane was added to C medium, and adjust pH to 9.0) (175 mL) in 250 mL plastic tissue culture flasks under the following culture conditions: 16 h light/8 h dark photo-cycle with light provided by LED light (30 µmole photons m^−2^ s^−1^) at 17 °C for 28 days. The cells were harvested by centrifugation at 4820× *g* for 15 min at 4 °C, suspended with 2.0 mL of 0.5 M AcOH, and sonicated three times for 30 s on ice. Then, the resulting solution was centrifuged at 4160× *g* for 15 min at 4 °C. The supernatant was adjusted to pH 7–8 using 2 M NH_3_ aq and loaded on an activated charcoal column (5 mL, FUJIFILM Wako Pure Chemical Industries, Ltd., Osaka, Japan). After the column was washed with water (15 mL), PSTs were eluted with AcOH-EtOH-H_2_O (5:50:45, *v/v/v*, 30 mL). The solvent was removed using a rotary evaporator under vaccum, and the resulting residue was resuspended with 1 mL of 0.05 M AcOH. This solution was filtered through a Cosmospin filter H (0.45 µm, Nacalai Tesque, Inc., Kyoto, Japan). A part of the filtrate was diluted depending on the concentrations of PSTs, and the diluted solution was applied to LCMS.

### 4.6. Harvest and Preparation of Alexandrium pacificum (Group IV) (120518KureAC) Cell Extract

*A. pacificum* (Group IV) (120518KureAC) was originally isolated at Kure, Hiroshima, Japan, by Dr. Kazuhiko Koike of Hiroshima University in 2012. It was maintained and grown in modified T_1_ medium prepared in artificial seawater as 200-mL cultures in 250 mL plastic tissue culture flasks under the following culture conditions: 12 h light/12 h dark photo-cycle with light provided by cool white bulbs (100–150 µmole photons m^−2^ s^−1^) at 15 °C. Aliquots of cultured cells (18 mL: 2.2 × 10^4^ cells mL^−1^) was centrifuged at 810× *g* for 3 min to pellet the cells. The pellets were re-suspended with 300 µL of 0.5 M acetic acid, and the cells were disrupted by sonication (3 times at 100 Hz for 30 s each, with an interval of 30 s) on ice. The suspension was centrifuged (20,000× *g* for 5 min at 4 °C), and the supernatant was subjected to ultra-filtration (10,000 Da cut-off, UF-MC). An aliquot (100 µL) of the extract after filtration was transferred to a new tube and mixed with three volumes of THF. The sample was loaded onto Chromabond^R^ HILIC (500 mg, MACHEREY-NAGEL, Düren, Germany) that had been pre-conditioned with 1 mL of MilliQ water and 5 mL of THF. Following loading of the sample, the column was sequentially washed with 3 mL of THF, 3 mL of CH_3_CN, and 3 mL of CH_3_CN-H_2_O-HCOOH (95:5:0.1, *v/v/v*) [33]. The column was eluted with 3 mL of 0.2 M HCOOH and concentrated under nitrogen stream. The volume was adjusted to 100 µL with MilliQ water and passed through a Cosmospin filter H (0.45 µm).

### 4.7. HR-RP-LCMS and HR-RP-LC-MS/MS Conditions for PSTs Analysis

HR-RP-LCMS was performed on an Inertsustain AQ-C18 column (4.6 i.d. × 250 mm, 5 μm, GL Sciences, Japan) with the mobile phase, HCOOH-H_2_O 0.1:100 (*v/v*). The flow rate was 0.2 mL/min. The injected volume was 1 µL. The oven temperature was 25 °C. HR-LCMS were recorded on a micrOTOF-Q II mass spectrometer (Bruker Daltonics) equipped with an ESI ion source. The liquid chromatography system used for analysis was a Shimadzu Nexera UHPLC System (Shimadzu). The mass spectrometer conditions were as follows: positive ionization mode; dry gas: nitrogen 7 L/min; dry heater temperature: 180 °C; nebulizer: 1.6 Bar; and capillary: 4500 V. Extracted ion chromatograms (EIC) were presented based on ± 0.02. HR-LC-MS/MS was performed in AutoMS/MS mode setting, with [M + H]^+^ as the precursor ions. The precursor ions were *m/z* 364.10 for 12β-deoxyGTX5 (**2**) and *m/z* 284.11 for 12β-deoxySTX (**3**) setting the width 3 Da. The sweeping collision energy was 40–60 eV for **2** and 34–52 eV for **3**.

## Figures and Tables

**Table 1:** ^1^H NMR (600 MHz) data for synthetic 12β-deoxyGTX5 (**2**), 12β-deoxySTX (**3**), and 12α-deoxyGTX5 (**5**).

	12β-deoxyGTX5 (12α-GTXol 5) (2) *		12β-deoxySTX (12α-STXol) (3) *		12α-deoxyGTX5 (12β-GTXol 5) (5) *	
No.	δ_H_	Multiplicity	δ_H_	Multiplicity	δ_H_	Multiplicity
		(*J* in Hz)		(*J* in Hz)		(*J* in Hz)
5	4.67	s	4.66	s	4.81	s
6	3.91	t 5.3	3.87	t 5.2	3.88	dd 9.6, 5.6
10α	3.74	t 9.8	3.75	t 9.7	3.77	t 10.0
10β	3.61	q 9.5	3.56	q 9.3	3.72	t 9.1
11α	1.98	m	1.99	m	2.25 **	dd 6.5, 12.1
11β	2.50	quint. 9.7	2.50	quint. 10.9	2.41 **	m
12	4.40	dd 7.6, 11.8	4.38	dd 7.6, 11.8	4.34	d 4.7
13a	4.33	m	4.25	dd 4.6, 11.8	4.39	t 9.1
13b	4.33	m	4.20	dd 6.4, 11.8	4.13	dd 5.9, 9.7

CD_3_COOD-D_2_O (4:96, *v*/*v*). The signal of CHD_2_COOD (2.06 ppm) was used as an internal reference. * This study. ** Interchangeable assignment.

## Data Availability

Not applicable.

## Supplementary Materials

The following supporting information can be downloaded at: https://www.mdpi.com/article/10.3390/md20030166/s1: Figure S1. The ESI-HRMS spectrum of synthetic 12β-deoxyGTX5 (**2**). Figure S2. The ESI-HRMS spectrum of synthetic 12β-deoxySTX (**3**). Figure S3. The ESI-HRMS spectrum of synthetic 12α-deoxyGTX5 (**5**). Figure S4. The COSY spectrum of synthetic 12β-deoxyGTX5 (**2**). Figure S5. The COSY spectrum of synthetic 12β-deoxySTX (**3**). Figure S6. The ^1^H NMR spectrum of synthetic 12α-deoxyGTX5 (**5**). Figure S7. The COSY spectrum of synthetic 12α-deoxyGTX5 (**5**). Figure S8. The TOCSY spectrum of synthetic 12α-deoxyGTX5 (**5**). Figure S9. The NOESY1D spectrum of synthetic 12β-deoxyGTX5 (**2**). Figure S10. The NOESY1D spectrum of synthetic 12β-deoxySTX (**3**). Figure S11. The ESI-HRMS spectrum of 12β-deoxyGTX5 (**2**) in *D. circinale* (TA04). Figure S12. The ESI-HRMS spectrum of 12β-deoxySTX (**3**) (α-saxitoxinol) in *D. circinale* (TA04). Figure S13. The ESI-HRMS spectrum of 12β-deoxySTX (**3**) (α-saxitoxinol) in *A. pacificum* (Group IV) (Kure AC). Figure S14. The HR-RP-LCMS Q1 scan of the *D. circinale* (TA04) cell extract.
