# Supplementary material for: First Identification of 12β-Deoxygonyautoxin 5 (12α-Gonyautoxinol 5) in the Cyanobacterium Dolichospermum circinale (TA04) and 12β-Deoxysaxitoxin (12α-Saxitoxinol) in D. circinale (TA04) and the Dinoflagellate Alexandrium pacificum (Group IV) (120518KureAC)
Source: Mar Drugs. 2022 Feb 25;20(3):166. doi: 10.3390/md20030166 (PMC8954441; doi:10.3390/md20030166)
Supplement: Supplementary file 1 [file marinedrugs-20-00166-s001.zip › marinedrugs-1560468-supplementary.pdf]

Supplementary Materials

First Identification of 12β-Deoxygonyautoxin 5 (12α-Gonyautoxinol) in the Cyanobacterium *Dolichospermum Circinale* (TA04), and 12β-Deoxysaxitoxin (12α- Saxitoxinol) in *D. Circinale* (TA04) and the Dinoflagellate *Alexandrium pacificum* (Group IV) (120518kureac)

Michiru Akamatsu[1,†], Ryosuke Hirozmi[1,†], Yuko Cho[1], Yuta Kudo[1,2], Keiichi Konoki[1], Yasukatsu Oshima[3,‡] and Mari Yotsu-Yamashita[1,*]

1 Graduate School of Agricultural Science, Tohoku University, Sendai 980-8572, Japan;

mg_rbbs9_0130@icloud.com (M.A.); ryousuke.hirozumi.t2@dc.tohoku.ac.jp (R.H.);

yuko.cho.a4@tohoku.ac.jp (Y.C.); yuta.kudo.d5@tohoku.ac.jp (Y.K.); keiichi.konoki.b2@tohoku.ac.jp (K.K.)

2 Frontier Research Institute for Interdisciplinary Sciences, Tohoku University, Sendai 980-8578, Japan

3 Graduate School of Life Sciences, Tohoku University, Sendai 980-8577, Japan; oshima.y3@gmail.com

* Correspondence: mari.yamashita.c1@tohoku.ac.jp; Tel.: +81-22-757-4425

† These authors contributed equally to this work.

‡ Professor emeritus.

Figure S1. The ESI-HRMS spectrum of synthetic 12β-deoxyGTX5 (**2**).
Figure S2. The ESI-HRMS spectrum of synthetic 12β-deoxySTX (**3**).
Figure S3. The ESI-HRMS spectrum of synthetic 12α-deoxyGTX5 (**5**).
Figure S4. The COSY spectrum of synthetic 12β-deoxyGTX5 (**2**).
Figure S5. The COSY spectrum of synthetic 12β-deoxySTX (**3**).
Figure S6. The ^{1}H NMR spectrum of synthetic 12α-deoxyGTX5 (**5**).
Figure S7. The COSY spectrum of synthetic 12α-deoxyGTX5 (**5**).

Figure S8. The TOCSY spectrum of synthetic 12α-deoxyGTX5 (**5**).
Figure S9. The NOESY1D spectrum of synthetic 12β-deoxyGTX5 (**2**).
Figure S10. The NOESY1D spectrum of synthetic 12β-deoxySTX (**3**).
Figure S11. The ESI-HRMS spectrum of 12β-deoxyGTX5 (**2**) in *D. circinale* (TA04).
Figure S12. The ESI-HRMS spectrum of 12β-deoxySTX (**3**) (α-saxitoxinol) in *D. circinale* (TA04).
Figure S13. The ESI-HRMS spectrum of 12β-deoxySTX (**3**) (α-saxitoxinol) in *A. pacificum* (Group IV) (Kure AC).
Figure S14. The HR-RP-LCMS Q1 scan of the *D. circinale* (TA04) cell extract.

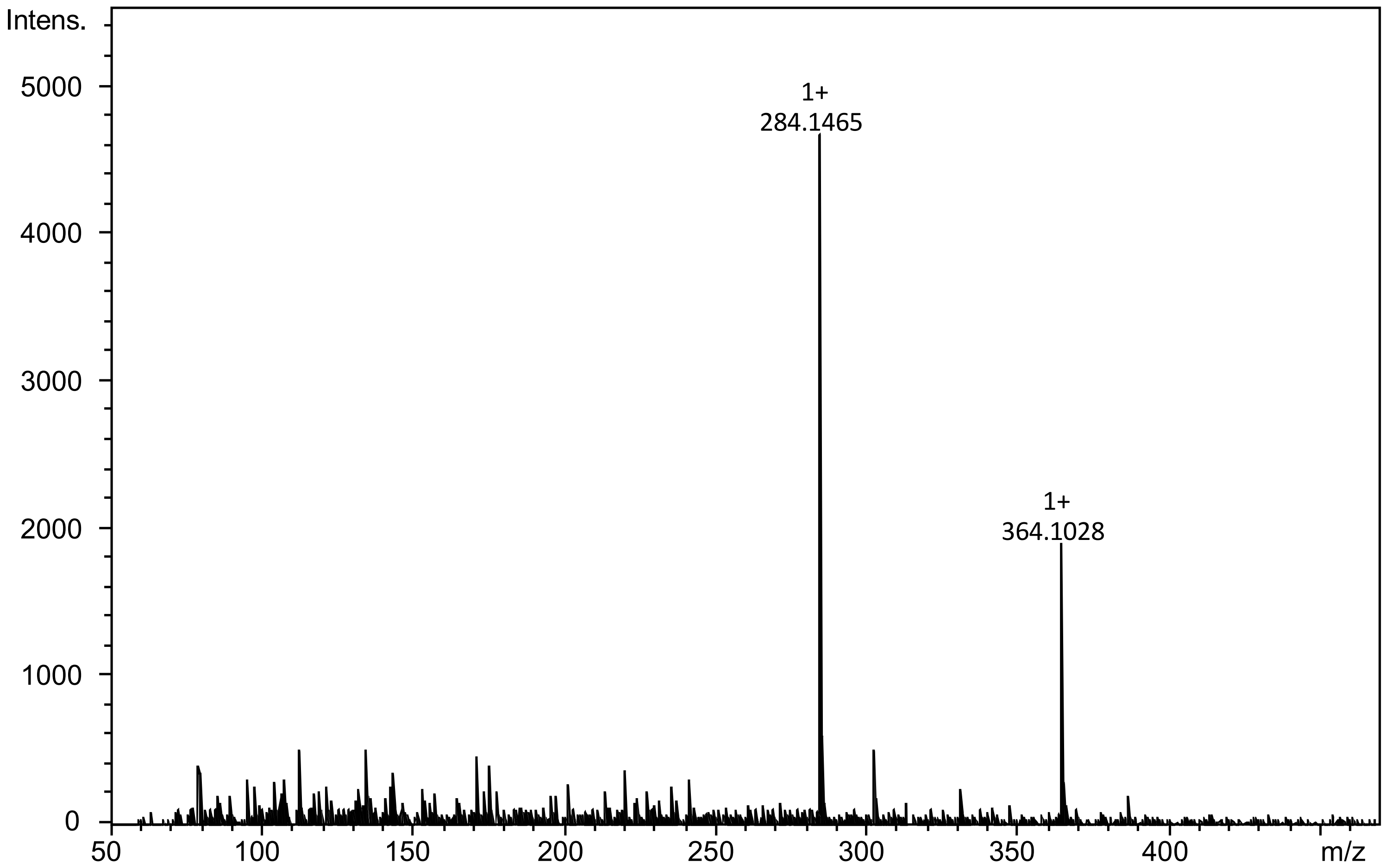

Figure S1. The ESI-HRMS spectrum of synthetic 12β-deoxyGTX5 (**2**).

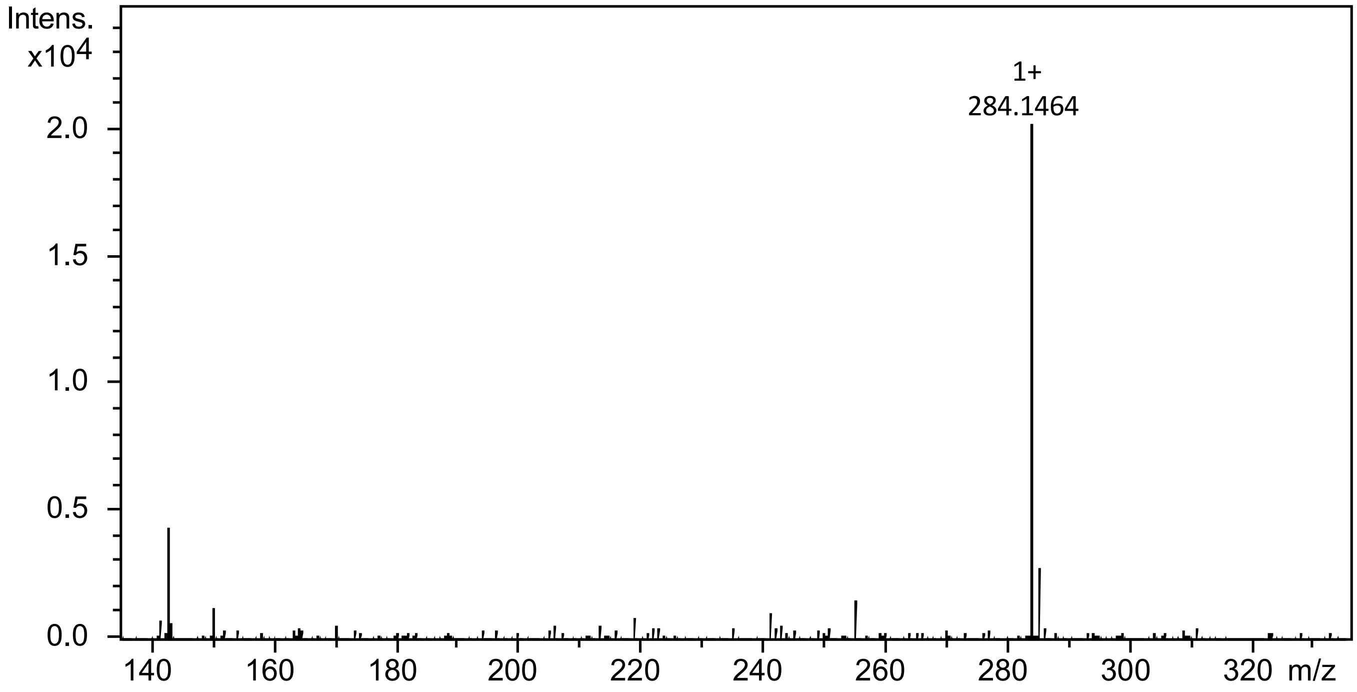

Figure S2. The ESI-HRMS spectrum of synthetic 12β-deoxySTX (**3**).

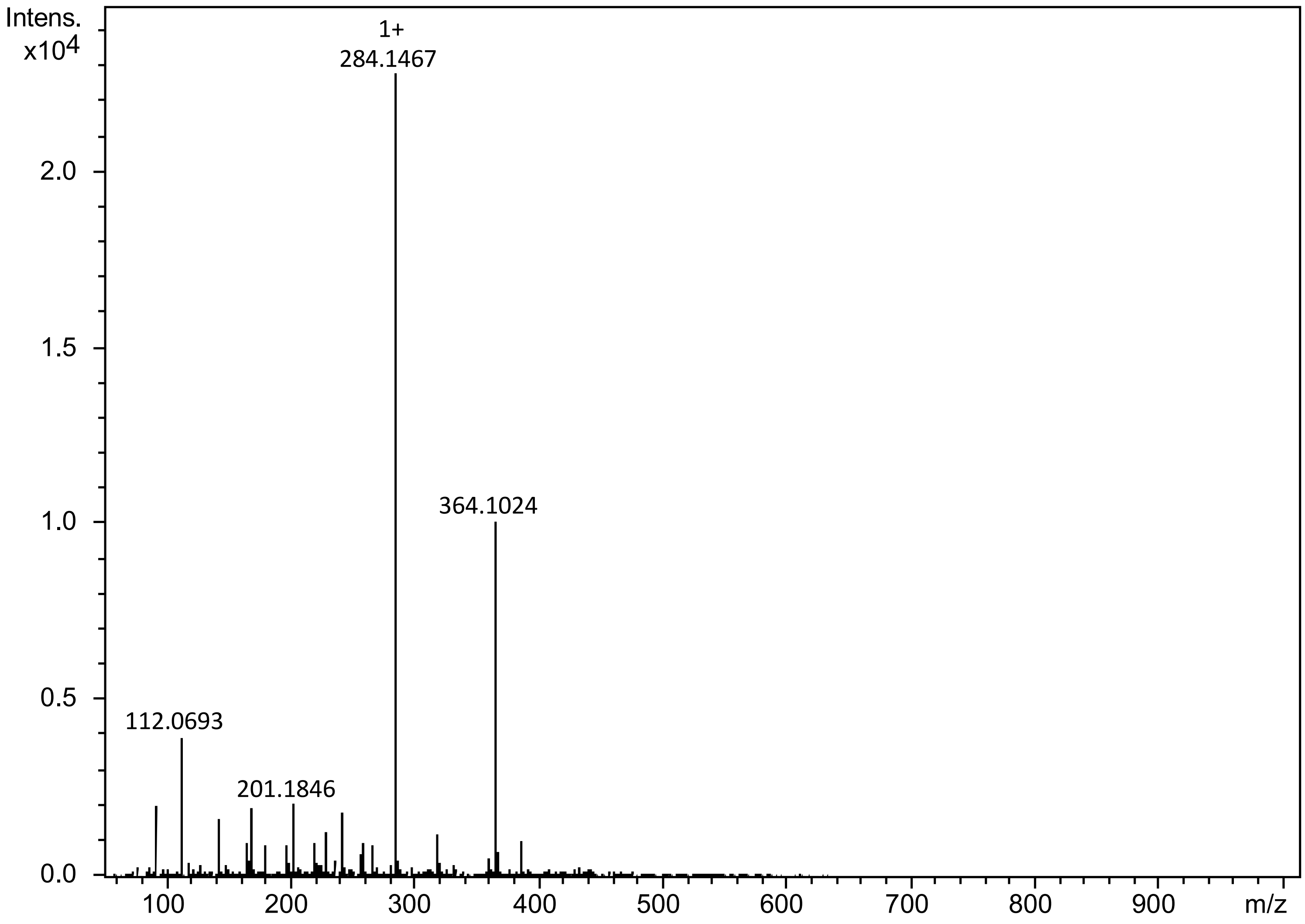

Figure S3. The ESI-HRMS spectrum of synthetic 12α-deoxyGTX5 (**5**).

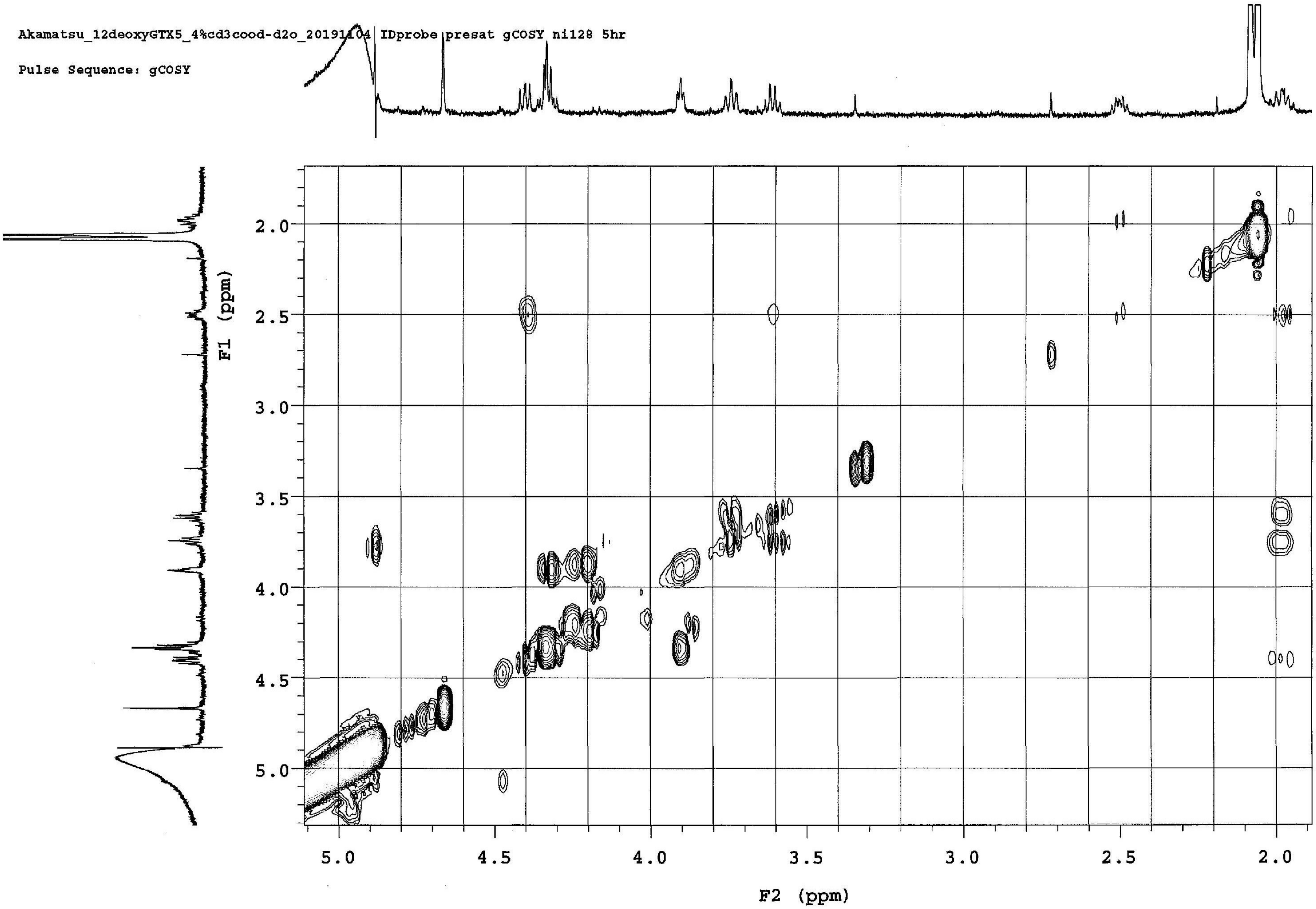

Figure S4. The COSY spectrum of synthetic 12β-deoxyGTX5 (**2**). (600 MHz, CD_3COOD-D_2O (4 : 96, v/v)).

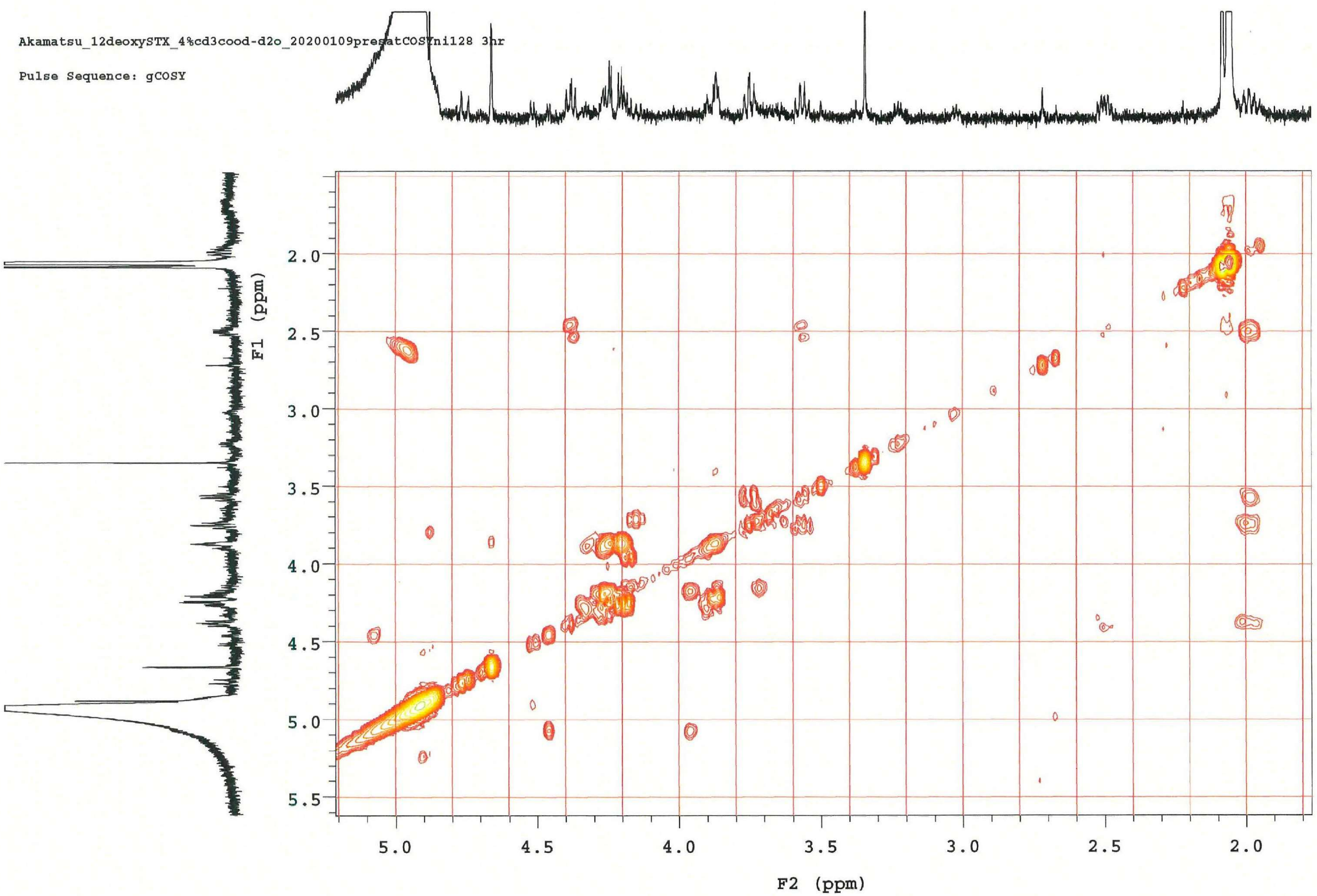

Figure S5. The COSY spectrum of synthetic 12β-deoxySTX (**3**).
(600 MHz, CD_3COOD-D_2O (4 : 96, v/v)).

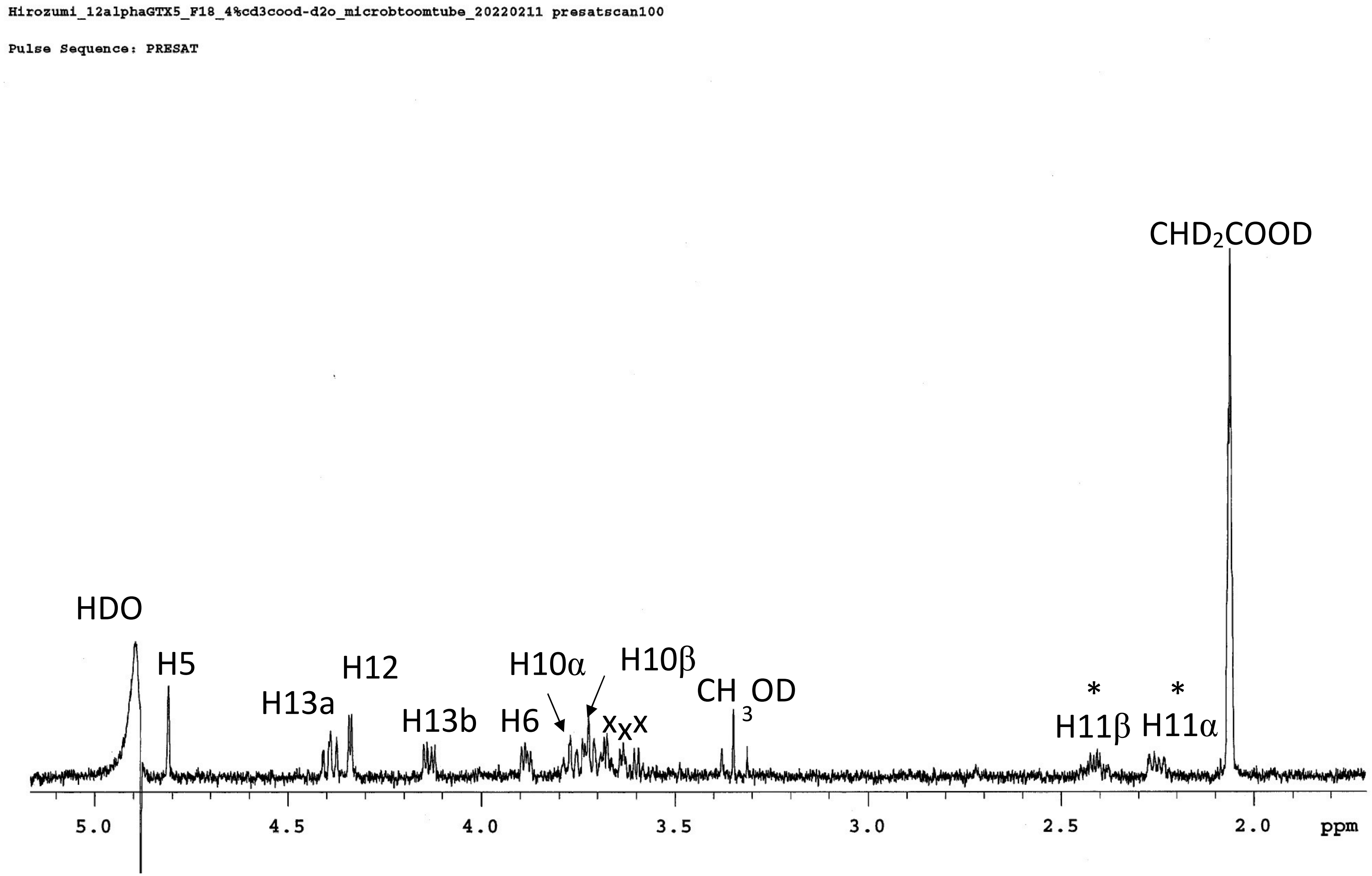

Figure S6. The ^{1}H NMR spectrum of synthetic 12α-deoxyGTX5 (**5**).
(600 MHz, microbottom tube, CD_3COOD-D_2O (4 : 96, v/v). HDO was presaturated.).
* interchangeable assignment.

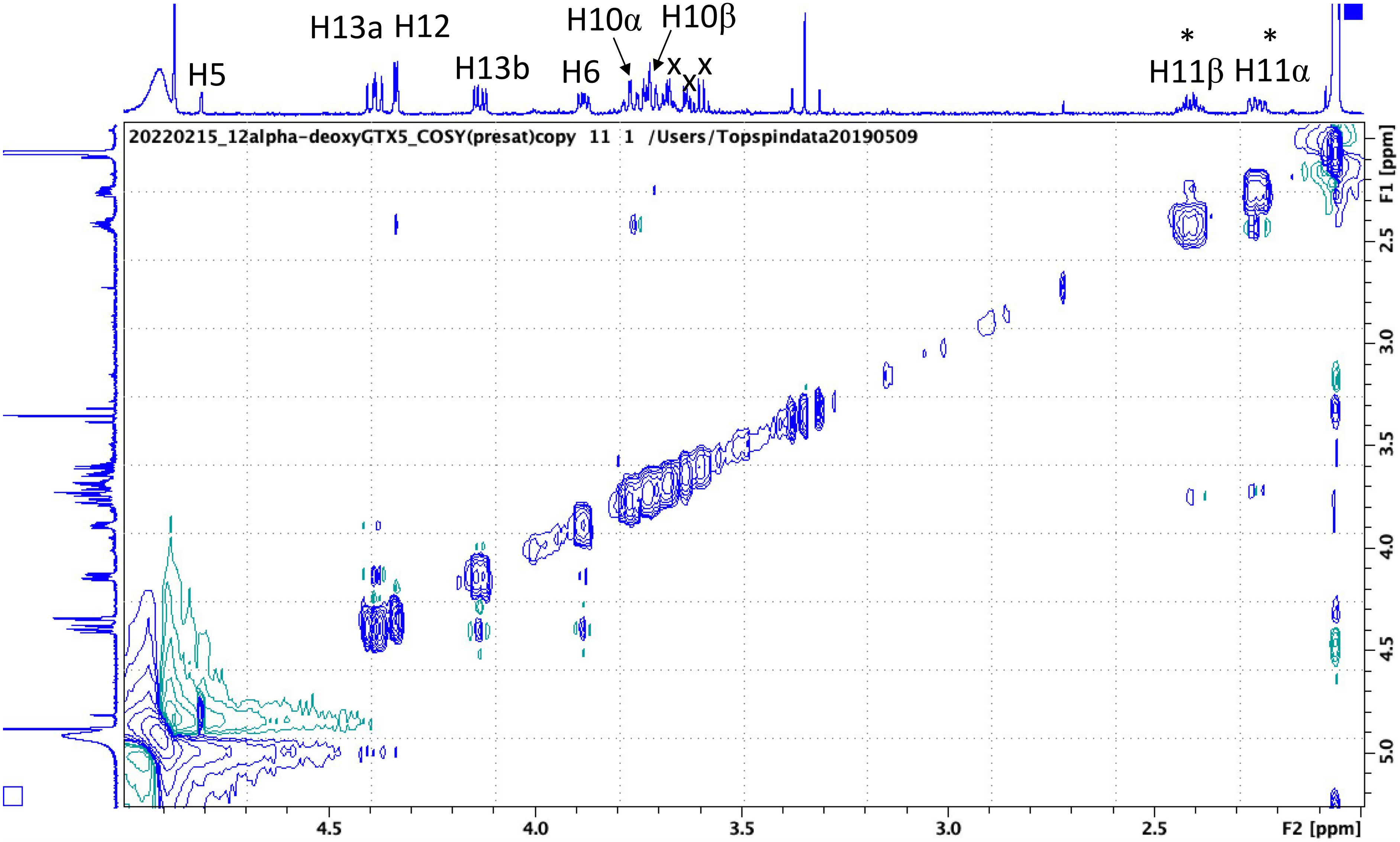

Figure S7. The COSY spectrum of synthetic 12α-deoxyGTX5 (**5**).
(600 MHz, microbottom tube, CD_3COOD-D_2O (4 : 96, v/v). HDO was presaturated. 1 h, Cryoprobe, Bruker AVANCE III), * interchangeable assignment.

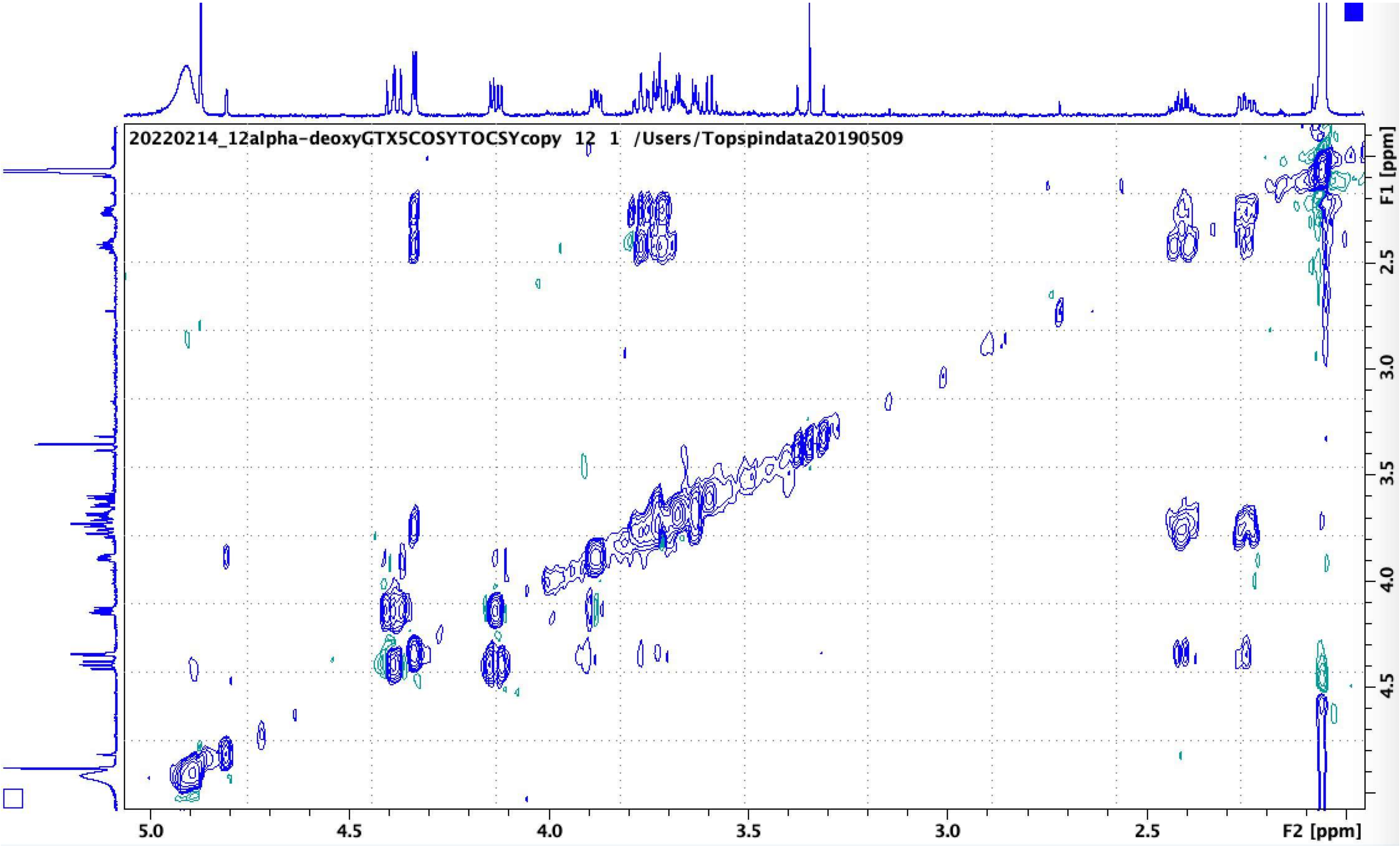

Figure S8. The TOCSY spectrum of synthetic 12α-deoxyGTX5 (**5**).
(600 MHz, microbottom tube, CD_3COOD-D_2O (4 : 96, v/v). HDO was presaturated. Cryoprobe, 10 min, Bruker AVANCE III)

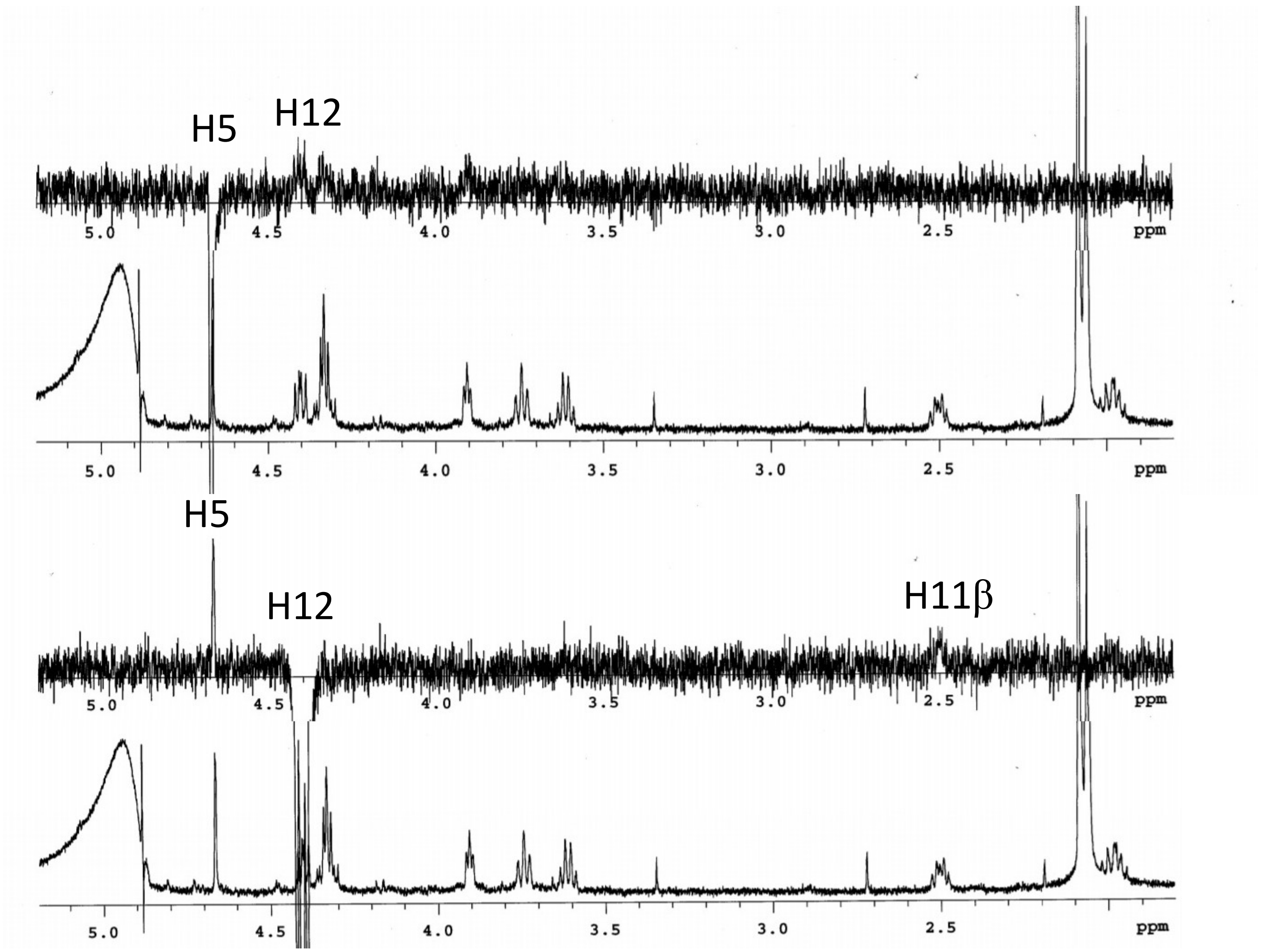

Figure S9. The NOESY 1D spectra of 12β-deoxyGTX5 (**2**) irradiate at δ_H 4.67 ppm (H5) and δ_H 4.40 ppm (H12) (600 MHz, $CD_3COOD-D_2O$ (4 : 96, v/v)).

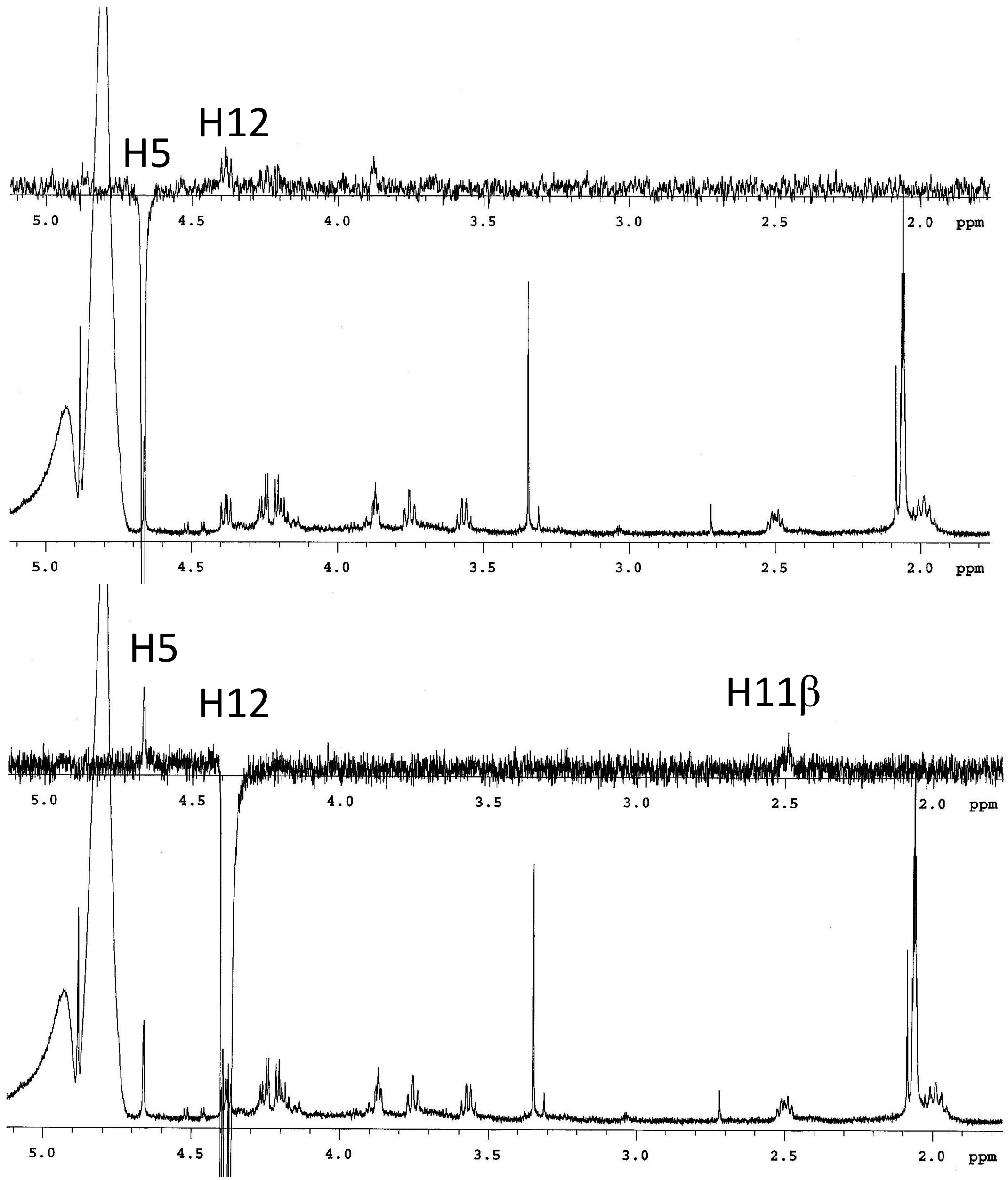

Figure S10. The NOESY 1D spectra of 12β-deoxySTX (**3**) irradiate at δ_H 4.66 ppm (H5) and δ_H 4.38 ppm (H12) (600 MHz, CD_3COOD-D_2O (4 : 96, v/v)).

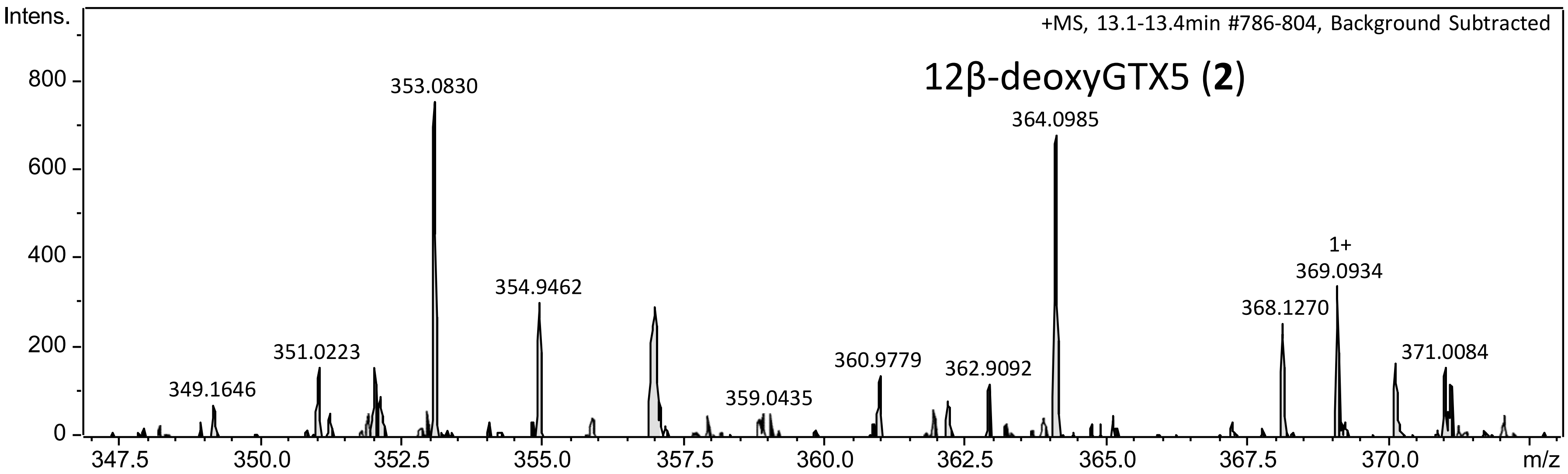

Figure S11. The ESI-HRMS spectrum of 12β-deoxyGTX5 (**2**) in *D. circinale* (TA04).

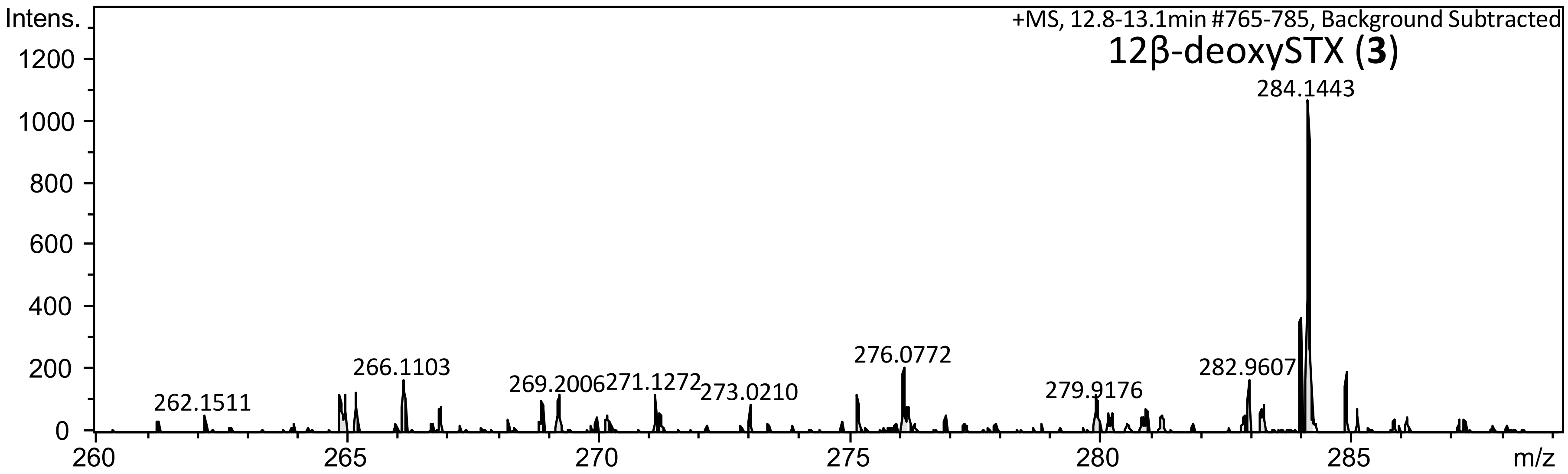

Figure S12. The ESI-HRMS spectrum of 12β-deoxySTX (**3**) (12α-saxitoxinol) in *D. circinale* (TA04).

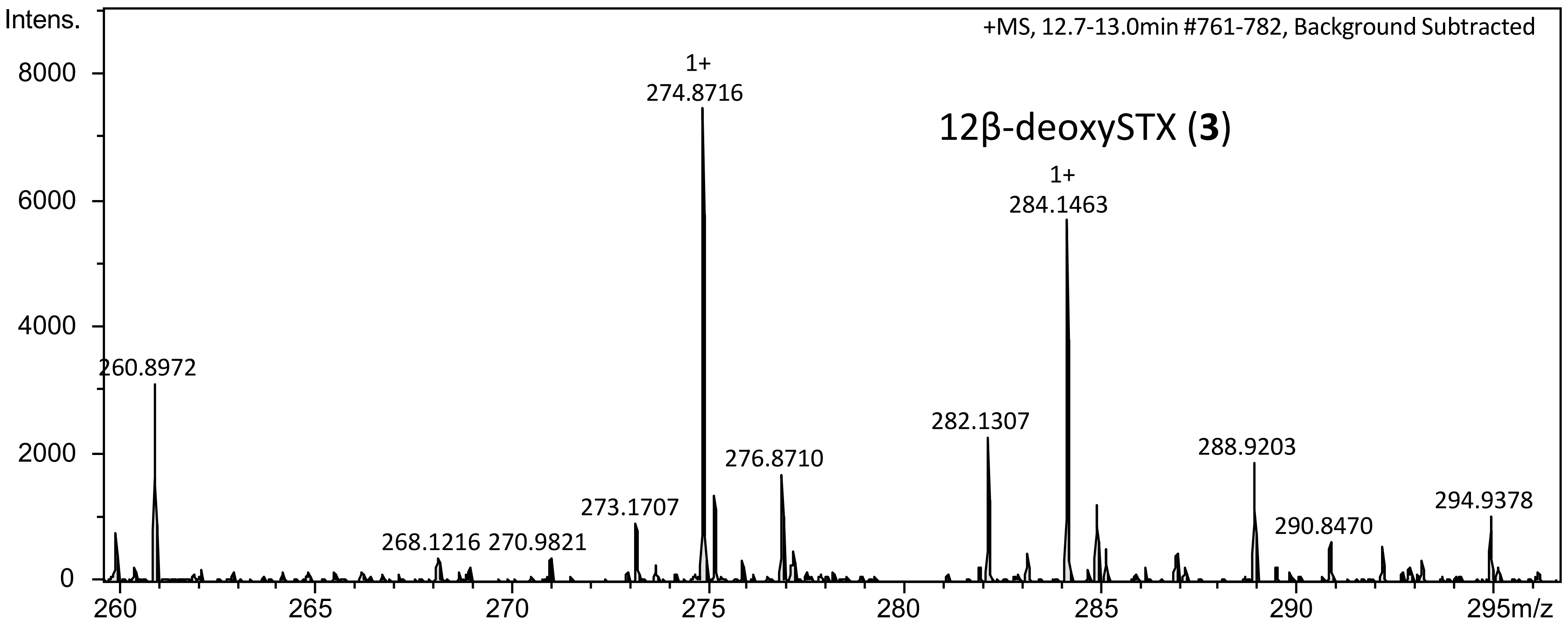

Figure S13. The ESI-HRMS spectrum of 12β-deoxySTX (**3**) (12α-saxitoxinol) in *A. pacificum* (Group IV) (Kure AC).

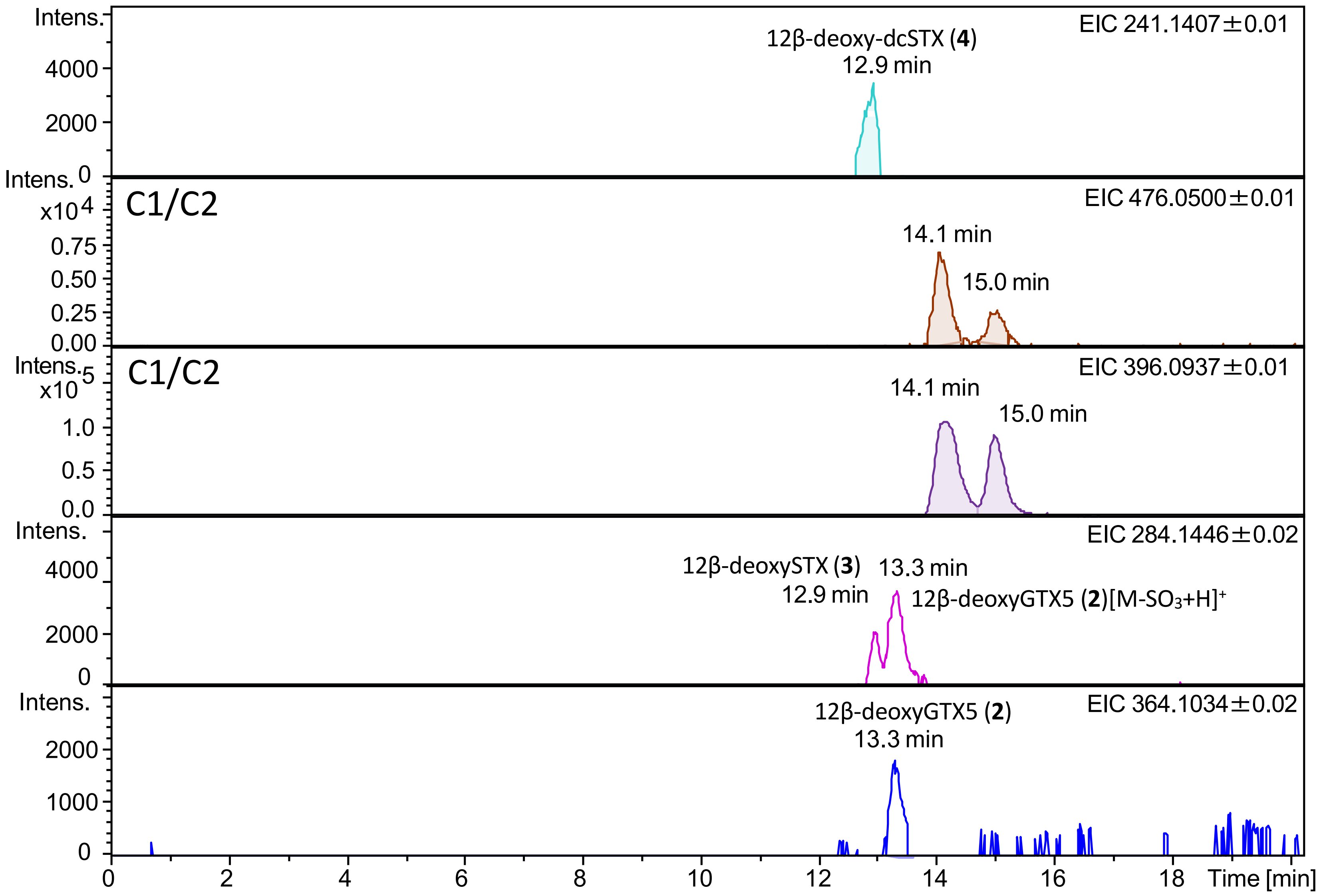

Figure S14. The HR-RP-LCMS Q1 scan of the *D. circinale* (TA04) cell extract. For LCMS condition, see text section 4.7.
